# Design and expression of a chimeric recombinant antigen (SsIR-Ss1a) for the serodiagnosis of human strongyloidiasis: Evaluation of performance, sensitivity, and specificity

**DOI:** 10.1371/journal.pntd.0012320

**Published:** 2024-07-15

**Authors:** Mostafa Omidian, Zohreh Mostafavi-Pour, Marzieh Asadi, Meysam Sharifdini, Navid Nezafat, Ali Pouryousef, Amir Savardashtaki, Mortaza Taheri-Anganeh, Fattaneh Mikaeili, Bahador Sarkari

**Affiliations:** 1 Student Research Committee, Shiraz University of Medical Sciences, Shiraz, Iran; 2 Recombinant Proteins Laboratory, Department of Biochemistry, School of Medicine, Shiraz University of Medical Sciences, Shiraz, Iran; 3 Department of Medical Biotechnology, School of Advanced Medical Sciences and Technologies, Shiraz University of Medical Sciences Shiraz, Iran; 4 Department of Medical Parasitology and Mycology, School of Medicine, Guilan University of Medical Sciences, Rasht, Iran; 5 Pharmaceutical Sciences Research Center, Shiraz University of Medical Sciences, Shiraz, Iran; 6 Department of Pharmaceutical Biotechnology, School of Pharmacy, Shiraz University of Medical Sciences, Shiraz, Iran; 7 Cellular and Molecular Research Center, Cellular and Molecular Medicine Research Institute, Urmia University of Medical Sciences, Urmia, Iran; 8 Basic Sciences in Infectious Diseases Research Center, Shiraz University of Medical Sciences, Shiraz, Iran; IRCCS Sacro Cuore Don Calabria Hospital, ITALY

## Abstract

**Background:**

The sensitivity of parasitological and molecular methods is unsatisfactory for the diagnosis of strongyloidiasis, and serological techniques are remaining as the most effective diagnostic approach. The present study aimed to design and produce a chimeric recombinant antigen from *Strongyloides stercoralis* immunoreactive antigen (*SsIR*) and Ss1a antigens, using immune-informatics approaches, and evaluated its diagnostic performance in an ELISA system for the diagnosis of human strongyloidiasis.

**Methodology/Principal findings:**

The coding sequences for *SsIR* and Ss1a were selected from GenBank and were gene-optimized. Using bioinformatics analysis, the regions with the highest antigenicity that did not overlap with other parasite antigens were selected. The chimeric recombinant antigen SsIR- Ss1a, was constructed. The solubility and physicochemical properties of the designed construct were analyzed and its tertiary structures were built and evaluated. The construct was expressed into the pET-23a (+) expression vector and the optimized DNA sequences of SsIR-Ss1a (873 bp) were cloned into competent *E*. *coli* DH5α cells. Diagnostic performances of the produced recombinant antigen, along with a commercial kit were evaluated in an indirect ELISA system, using a panel of sera from strongyloidiasis patients and controls.

The physicochemical and bioinformatics evaluations revealed that the designed chimeric construct is soluble, has a molecular with of 35 KDa, and is antigenic. Western blotting confirmed the immunoreactivity of the produced chimeric recombinant antigen with the sera of strongyloidiasis patients. The sensitivity and specificity of the indirect ELISA system, using the produced SsIR-Ss1a chimeric antigen, were found to be 93.94% (95% CI, 0.803 to 0.989) and 97.22% (95% CI, 0.921 to 0.992) respectively.

**Conclusions/Significance:**

The preliminary findings of this study suggest that the produced SsIR-Ss1a chimeric antigen shows promise in the diagnosis of human strongyloidiasis. However, these results are based on a limited panel of samples, and further research with a larger sample size is necessary to confirm its accuracy. The construct has potential as an antigen in the ELISA system for the serological diagnosis of this neglected parasitic infection, but additional validation is required.

## Author summary

In our study, we aimed to improve the diagnosis of strongyloidiasis, a parasitic infection that is often difficult to detect with current methods. Traditional parasitological and molecular techniques have limitations in sensitivity, making it challenging to accurately diagnose this condition. To address this, we designed a new diagnostic tool using a chimeric recombinant antigen, combining two specific antigens from the parasite *Strongyloides stercoralis*. We used advanced bioinformatics to select the most immunogenic regions of these antigens, ensuring they did not overlap with other parasite proteins. This chimeric antigen was then produced and tested for its diagnostic performance in an ELISA system. Our results showed that the new chimeric antigen is both soluble and highly antigenic; meaning it effectively triggers an immune response. When tested with sera samples from patients with strongyloidiasis, the ELISA system using our chimeric antigen demonstrated high sensitivity (93.94%) and specificity (97.22%), indicating it could accurately identify infected individuals.

These findings are promising, suggesting that our chimeric antigen could be a valuable tool for diagnosing strongyloidiasis. However, further research with a larger sample size is needed to confirm its effectiveness. If validated, this new diagnostic approach could significantly improve the detection and management of this neglected parasitic infection, benefiting both patients and healthcare providers.

## Introduction

Strongyloidiasis is a neglected tropical disease caused by the soil-transmitted nematode *Strongyloides stercoralis (S*. *stercoralis)*, which affects around 600 million people in tropical and subtropical areas [1,2]. The infective stage known as filariform larvae penetrates the skin and enters the lungs via the bloodstream. They migrate up to the airways, swallowed, and reach the small intestine where they become adults following molting [3]. Approximately half of infections are asymptomatic, but people may complain of non-specific gastrointestinal disorders, respiratory disorders such as wheezing, itching, and larva currens [4]. Its peculiar auto-infective cycle causes chronic, probably life-long infection, which can develop into frequently fatal hyper infection syndrome in immunocompromised individuals, hence prompt diagnosis is crucial [5]. Currently, there are various methods for detecting strongyloidiasis, including stool examination, molecular approaches, and immunodiagnostic assays. Direct stool examination has limited sensitivity, with a single sample yielding positive results in up to 50% of cases [6–8]. The Baermann technique, which requires multiple samples for sufficient sensitivity, is a complex and inaccessible test in most clinical laboratories. Agar plate culture can improve sensitivity but is time-consuming and labor-intensive [9]. Molecular methods have shown varying sensitivity levels depending on the infection burden [10,11]. Based on parasitological tests as references, the average sensitivity of molecular techniques is 71.8% [12].

Parasitological and molecular approaches have low sensitivity in comparison with the serological tests, especially in low-burden intestinal parasitic infections. The enzyme-linked immunosorbent assay (ELISA) has become the preferred choice among serological techniques due to its simplicity and availability in most diagnostic laboratories. Several ELISA systems evaluated for the diagnosis of strongyloidiasis have shown higher sensitivity in the diagnosis of strongyloidiasis compared to fecal-based tests [13,14]. Also, it is useful for screening of the disease in strongyloidiasis-endemic areas [7,15].

The source of the antigen significantly impacts test performance. ELISA based on crude antigens has drawbacks, including constant dependence on a parasite source and batch-to-batch variation [13,14]. On the other hand, recombinant proteins are highly purified and can be produced in large quantities to replace crude parasite extracts [16].

So far, several recombinant antigens have been produced and evaluated for the diagnosis of strongyloidiasis, of which a 31-kDa recombinant antigen (NIE), *S*. *stercoralis* immunoreactive antigen (SsIR), fatty acid and retinol-binding protein (FAR), 14-3-3 protein, and the recently introduced Ss1a-recombinant protein have received more attention [17–20]. Despite the progress achieved in recent years in the field of serological diagnosis of strongyloidiasis, there are still various challenges in the diagnosis of this neglected parasitic disease. Hence, it seems necessary to identify, design, and produce new antigens with appropriate efficiency in the diagnosis of this disease. In this regard, the present study aimed to design and produce a chimeric recombinant antigen from SsIR and Ss1a antigens, using immune-informatics approaches, and tested its diagnostic performance in an ELISA system for the diagnosis of human strongyloidiasis in the clinical setting.

## Materials and methods

### Ethics statement

The Ethics Committee of Shiraz University of Medical Sciences (SUMS) approved the research protocols (Ref. No. R.SUMS.REC.1398.1170). Formal consent (verbal) was obtained from the participants, or from the parent/guardian in case of children.

### Bioinformatics analysis

#### Selection of antigens and sequence retrieval

**Fig 1:**
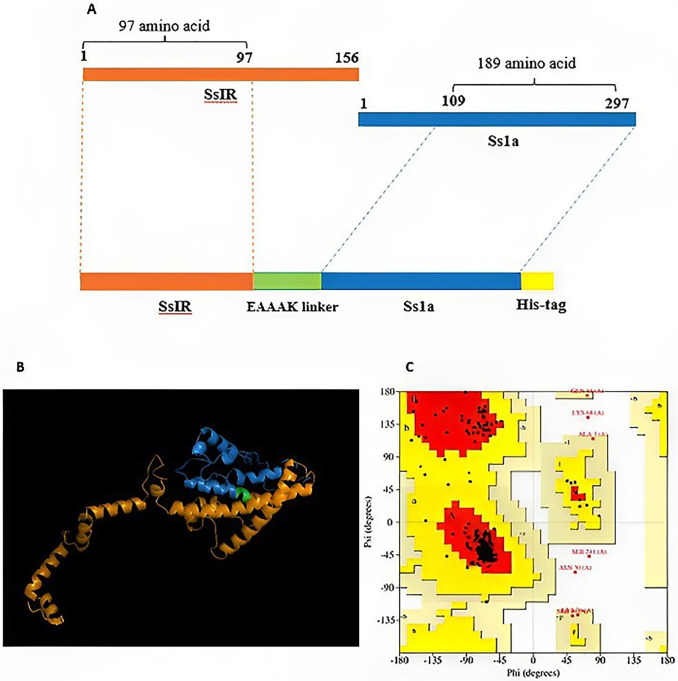
Construction, tertiary structure prediction and validation of the chimeric antigen. **(A)** Schematic presentation of the chimeric antigen construct, comprising SsIR (in orange), Ss1a (in blue), connected by EAAAK linker (in green), and a 6xHis tag (in yellow) **(B)** Three-dimensional (3D) model of the chimeric antigen. **(C)** PROCHECK’s Ramachandran plot demonstrates that 92.6% of residues are in the allowed region, with 5.8% in the favored region.

The amino acid sequence of the SsIR (Accession No: O44394/ amino acid: 1–97) was obtained from the UniProt database (https://www.uniprot.org/) in FASTA format. Subsequently, the FASTA sequence of Ss1a was retrieved from the patent (Patent ID: WO2017091059A1/ amino acid: 109–297). After that, these sequences were joined using an EAAAK linker (Fig 1).

#### Physicochemical properties and solubility assessment

The physicochemical properties and solubility of the chimeric antigen were evaluated using the ProtParam tool, accessible on the EXPASY server (http://www.expasy.org/tools/protparam.html), and SolPro (https://scratch.proteomics.ics.uci.edu/), respectively. The ProtParam tool provided information about the amino acid (aa) amount and composition, predicted isoelectric point (pI), molecular weight (MW; KDa), stability index, aliphatic index, and Grand average of hydropathicity (GRAVY). SolPro is a machine-learning algorithm that analyzes protein solubility based on amino acid content, hydrophobicity, secondary structure, solvent accessibility, and charge [21,22].

#### Antigenicity and cross-reactivity analysis

The antigenicity of the chimeric antigen was predicted using the VaxiJen v2.0 server (http://www.ddgpharmfac.net/vaxijen/VaxiJen/VaxiJen.html), which calculates protein antigenicity. A threshold score of 0.4 was used, and VaxiJen’s accuracy ranges from 70% to 89%, depending on the target organism. [23–25]. Cross-reactivity was analyzed by selecting a list of pathogens or closely related organisms with similar epitopes and checking for cross-reactivity using BLAST-p (https://blast.ncbi.nlm.nih.gov/Blast.cgi). Initial evaluation of the complete sequences of the antigens revealed some commonalities with the epitopes of few parasites (e. g. *Fasciola spp* and *Plasmodium spp*), those regions which had no commonalities were selected for the designing of the chimeric antigen.

#### Tertiary structure prediction

The tertiary structure of the chimeric antigen was predicted using the GalaxyTBM service (https://galaxy.seoklab.org/cgi-bin/submit.cgi?type=TBM), which employs template-based modeling (TBM) [26]. The quality of the 3-dimensional (3D) structures was assessed using multiple web servers such as the PROCHECK server (https://saves.mbi.ucla.edu/) [27].

#### Codon optimization and in-silico cloning

In-silico cloning allows researchers to manipulate DNA sequences in a virtual environment before actually cloning DNA sequences in a laboratory. This technique involves using bioinformatics tools and algorithms to identify potential restriction enzyme sites. It also involves designing primers for PCR amplification, predicting the size of DNA fragments, and analyzing the sequence data. By simulating the cloning process computationally, researchers can save time and resources by optimizing their experimental design before conducting actual experiments.

To optimize the codon usage for efficient expression of the chimeric antigen in the desired host, the protein sequence was translated into DNA sequences using EMBOSS server. The Gene Smart Codon Optimization tool was used for codon optimization, available at http://www.genscript.com/gensmart-free-gene-codon-optimization.html. Subsequently, the rare codon analysis tool (https://www.genscript.com/tools/rare-codon-analysis) was employed to evaluate codon quality based on parameters such as codon adaptation index (CAI), optimal codon frequency (FOP), and GC content. Finally, the chimeric antigen was inserted into the pET-23a (+) vector under restriction sites *NdeI* and *XhoI*, using SnapGene v5.1.4.1 software.

#### Recombinant plasmid construction and cloning

The optimized chimeric antigen DNA sequence was then synthesized (GenScript, USA) and inserted at the *NdeI / XhoI* restriction site in the pET-23a (+) expression vector (Novagen, Madison, WI, USA). The recombinant construct (pET23a-SsIR-Ss1a) was transformed into competent *E*. *coli* DH5α cells (Novagen, USA), using the heat shock technique [28]. The related SsIR-Ss1a was sequenced (S1, Sequence of the cloned antigen (rSsIR-Ss1a)) and used to verify the cloning accuracy of the plasmid isolated from transformants (GenScript, USA).

#### Expression and purification of the chimeric recombinant antigen

Competent *E*. *coli* BL21 (DE3) cells (Novagen, USA) were transformed with a recombinant plasmid (pET23-SsIR-Ss1a) and the transformed cells were cultured at 37°C in 200 mL of Luria Broth (LB) media containing 200 μg/mL ampicillin. When achieving a cell density of 0.6, 1 mM isopropyl-b D-thiogalactopyranoside (IPTG) was added to the cultures, and then incubated for 6 hours at 37°C. Cells were then extracted by centrifugation at 6000 g for 15 minutes at 4°C. Bacterial pellets were resuspended and homogenized for 45 minutes at 4°C in 3 ml lysis buffer (50 mM Na_2_HPO_4_, 300 mM NaCl, 10 mM imidazole; pH: 8). Under cold conditions, the bacterial pellets were sonicated three times at 50% intensity for 30 seconds with 20 second intervals. The lysate was centrifuged at 12,000g for 30 minutes at 4°C. For 2 hours at 4°C, supernatants were applied to Ni-NTA resin (Qiagen, Hilden, Germany). In the next step, the resin was washed twice with 3 ml wash buffer (50 mM NaH_2_PO_4_, 25 mM imidazole, and 300 mM NaCl; (pH: 8). To wash away the specifically bound protein from the resin, 1 ml of elution buffer was used (50 mM NaH_2_PO_4_, 250 mM imidazole, 300 mM NaCl; pH:8). Finally, a BCA protein assay kit (Thermo Fisher Scientific, USA) was used to measure the protein concentration.

### SDS–PAGE and western blotting

The SsIR-Ss1a antigen was purified, and 10 μg of it was electrophoresed and confirmed by Western blotting. As a control, recombinant nucleocapsid N protein of SARS-CoV-2 was administered under the same conditions, as previously described by Savardashtaki et al.[29].

#### Serum sampling

Archived cryopreserved sera, which were obtained from patients who gave consent for the scientific use of leftover serum from routine analyses were used to examine the performance of SsIR-Ss1a chimeric antigen in the serodiagnosis of human strongyloidiasis. A total of 141 serum samples were divided into three groups: group A included 33 samples from individuals who tested positive for strongyloidiasis through parasitological or molecular methods. The majority of the positive samples (30 out of 33) were provided by our esteemed colleagues from the Parasitology Departments at Fasa and Guilan University of Medical Sciences, Iran. Of these 33 positive samples, 25 cases were confirmed by agar plate culture. Also, *S*. *stercoralis* infection was confirmed in eight cases by employing a nested PCR to amplify the cytochrome c oxidase subunit 1 (cox1) gene as described by Sharifdini et al. [10]. The second group (group B) consisted of 52 serum samples collected from patients with other parasitic diseases, including toxocariasis (N = 12), fascioliasis (N = 8), hydatidosis (N = 8), trichostrongylosis (N = 5), hymenolepiasis (N = 1), malaria (N = 4), toxoplasmosis (N = 3), visceral leishmaniasis (N = 1), giardiasis (N = 2), cryptosporidiosis (N = 1), fever of unknown origin (FUO) (N = 3), and autoimmune diseases (N = 4). The samples used for possible cross-reactivity were confirmed through parasitological and/or serological techniques (ELISA) for each particular infection. The reason for using malaria patients’ serum for cross reactivity was due to preliminary BLAST evaluations of the antigen sequences for constructing the chimeric antigen, which revealed a few common amino acid sequences. We hypothesized that these common sequences might cause cross-reactivity in our system, which was not observed. For patients with fever of unknown origin (FUO), hypergammaglobulinemia is often a feature, potentially increasing the chance of cross-reactivity in serological tests. This is why serum from these patients was included in the study. Possible co-infection was excluded in a few samples where stool samples were available. Additionally, samples were collected from areas where *S*. *stercoralis* is not endemic, reducing the likelihood of co-infection.

Group C consisted of 56 sera from individuals without a history of parasitic disease, referred to as negative healthy controls. For the negative control group, samples were tested for *Strongyloides* infection using both agar plate culture and ELISA methods. A sample was considered negative only when the results from both tests were negative.

#### Evaluation of the chimeric antigen by indirect ELISA

An indirect ELISA was developed to measure the anti-SsIR-Ss1a antibodies in the aforementioned sera samples. The antigen was prepared at a concentration of 1 μg/mL in coating buffer (0.5 M bicarbonate at pH 9.6) and pre-coated onto 96-well plates (Nunc, Denmark). The plates were then incubated overnight at 4°C. Afterward, the wells were rinsed three times with PBST and blocked with 5% skimmed milk/PBS-T for 90 minutes at 30°C. Subsequently, the wells were washed five times with PBS-T. A diluted serum (1:100) was added to each well (100 μl), and the plates were incubated for 90 minutes at 30°C. Following this, the wells were washed five times with PBST before adding HRP-conjugated anti-human antibody (Sigma-Aldrich, USA) at a volume of 100 μl. After an additional hour of incubation at 30°C, the plate was washed five times with PBS-T. Subsequently, orthophenyl-diaminobenzidine (OPD) substrate (100 μl) was added, and the process was concluded by adding H_2_SO_4_ (100 μl) to each well after a 15-minute incubation period. An ELISA reader (ELX800, BioTek, USA) was used to measure the optical density (OD) at a wavelength of 450 nm compared to the reference wavelength of 630 nm.

#### Western blotting for the detection of anti-SsIR-Ss1a antibodies

The purified SsIR-Ss1a antigen (10 μg) underwent electrophoresis on an 18% SDS-PAGE gel and subsequently transferred onto a nitrocellulose membrane with a pore size of 0.2 μm. The membrane was then blocked using a solution consisting of 5% skimmed milk diluted in TBS-T (Tris-buffered saline containing 0.05% Tween-20), which lasted for 90 minutes at 37°C. After cutting the membrane into strips, they were washed three times and incubated for another 90 minutes with a panel of sera including positive samples (N = 5), negative samples (N = 5), and other sera from non-strongyloidiasis patients (N = 5). Following three washes with TBS-T, the strips were incubated with secondary antibodies diluted at a ratio of 1: 3,000, goat anti-human IgG for another period of 90 minutes. Subsequently, the strips underwent three additional washes before being treated with 3,3’-Diaminobenzidine (DAB) solution (DAB from Sigma-Aldrich USA) to visualize immunoreactions.

### Evaluation of the samples by commercial ELISA Kit

Serum samples were evaluated using a commercial ELISA kit (NovaLisa, NovaTech, Germany) following the manufacturer’s instructions.

The cut-off was calculated based on the kit’s instructions. Briefly, the mean absorbance value of the Cut-Off Controls was measured, and using these ODs, the NovaTec Units (NTU) were determined. According to the kit’s guidelines, NTU values > 11 were considered positive, those between 9–11 were considered equivocal, and NTUs < 9 were considered negative. For equivocal samples, we repeated the test as per the kit’s instructions, and if the result remained equivocal, the sample was considered negative. Using Youden’s index, the optimal cut-off was obtained to make the results of the commercial ELISA and those of SsIR-Ss1a-ELISA comparable.

This kit utilizes a recombinant antigen and offers a sensitivity of 89.47% and a specificity of 94.12%.

#### Statistical analysis

As the serum samples were prepared, we conducted a post-hoc power analysis to estimate the marginal error of the estimated sensitivity/specificity. The results revealed that with the acquired samples and 80% of power, a 10% marginal error in the estimations is expected (unadjusted for the disease prevalence) [30].

GraphPad Prism (version 6.0, GraphPad Software, CA, USA) and SPSS (version 20, SPSS, Chicago, IL, USA) were used to analyze the data. Based on receiver-operating characteristic (ROC) curve analysis, the cut-off value for ELISA was determined with a 95% confidence interval (CI). A Mann–Whitney test was applied to determine differences between the experimental groups. P values of less than 0.05 were considered statistically significant. Cohen’s Kappa coefficient test was used to evaluate the agreement between the tests. The test’s evaluation part of this study is reported according to STARD recommendations (S1 STARD Checklist).

## Results

### Evaluation of physicochemical properties, antigenicity, and cross-reactivity

The physicochemical properties of the developed antigen were evaluated using Protparam data, which showed a molecular weight of 35 kDa and an isoelectric point of 6.65. The GRAVY and aliphatic index were calculated to be -1.062 and 76.74, respectively. Solpro indicated that the antigen is soluble, with a score of 0.637123. Additionally, Vaxijen results revealed that the chimeric antigen is antigenic, with a score of 0.6258, and does not display cross-reactivity with other parasitic diseases (Table 1).

**Table 1:** Evaluation of physicochemical properties, antigenicity, and cross-reactivity of chimeric antigen.

Sequence	Cross- reacting	Antigenicity	Physicochemical properties					
			aa	MW (kDa)	pI	Aliphatic index	GRAVY	Solubility
NSARVENQDQKDQLENQDQKDQLENQDQKNQLKNQSENQDQKNQLKNQSENQDQKKPIKKPIKKPGPKPIRPIVKPKPKTTTQAPEEPEGPEEPEGPEAAAKNLHLYYIIDFVLPWLKDKEESSSGPSISKDDKLTPSERRERILKRHQMYKNFEEKLLEYENEASTAGGLDDITQRNYVLAKLRTYALKAMMDLEKIGEELGILEYMLKIKQGEVVEEKHKPPPKMTTYRIVRNEEQKKSLEWVIKIFQHLLWMSGIVKWIQKDILILNRTPEHSPIPQIMEETMMMMII	No significant similarity	0.625 **(Antigen)**	291	34	6.65	76.74	-1.06	0.6371 **(Soluble)**

**Abbreviations:** amino acid (aa); Molecular Weight (MW); Isoelectric Point (pI); Grand Average of Hydropathy (GRAVY).

### Prediction of 3D structure

To predict the 3D structure, GalaxyTBM generated five models, and Ramachandran plots were used to compare these models. Model 1 was chosen as the superior 3D model based on Ramachandran’s plots (Fig 1 and Table 2).

**Table 2:** The high-score 3D models obtained from four designed sequences by GalaxyWeb server.

3D models			Model l a	Model 2	Model 3	Model 4	Model 5
**Validation method**	Ramachandran plot	Favored %	**92.6%**	92.2%	91.8%	91.1%	91.4%
		Generously allowed%	**5.8%**	5.8%	5.4%	6.6%	5.8%
		Additional allowed%	**1.2%**	1.2%	1.6%	0.8%	1.6%
		Disallowed %	**0.4%**	0.8%	1.2%	1.6%	1.2%

a: The best 3D model among all modeled sequences (Model 1) is in bold font.

#### Codon optimization and in-silico cloning

**Fig 2:**
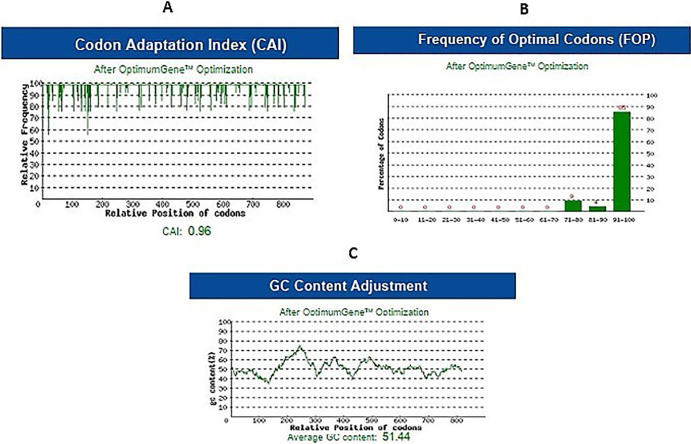
Codon optimization and secondary structure prediction of designed multi-epitope antigen. **(A)** CAI value (0.96%), **(B)** FOP value (85%), **(C)** GC content (51.44%). **Abbreviation:** Codon Adaptation Index (CAI); Frequency of Optimal Codons (FOP).

**Fig 3:**
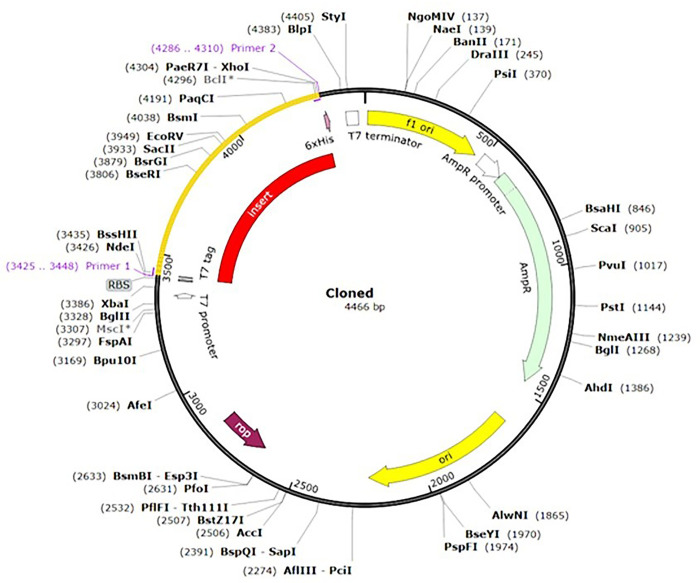
Representation of the in-silico cloning of the chimeric antigen constructs. The codon-optimized gene sequence of the chimeric antigen (represented in red and yellow) was cloned between the *NdeI* and *XhoI* restriction sites of the pET23a (+) expression vector (shown as a black circle).

The chimeric antigen was optimized through codon optimization and in-silico cloning to ensure efficient expression. The optimized sequence was analyzed using the Rare Codon Analysis tool, which showed a high degree of gene expression with a CAI value of 0.96%. The FOP value of 85% indicated the highest frequency of codon usage for each amino acid in the target expression organism (Fig 2A and 2B). The GC content (51.44%) was within the recommended range of 30–70% (Fig 2C). Recognition sites for restriction enzymes (*NdeI* and *XhoI*) were introduced at the end of the optimized sequence. Subsequently, the modified sequence was incorporated into a pET-23a (+) vector using SnapGene software (Fig 3).

### Construction, expression, and purification of the chimeric antigen

**Fig 4:**
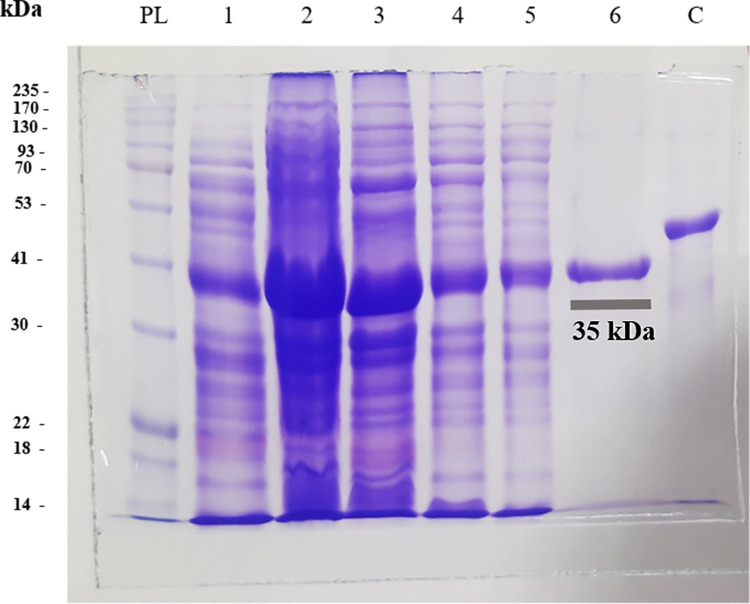
SDS-PAGE analysis of the recombinant chimeric antigen, analysis of different stages of protein purification. PL: protein Ladder; lane 1: non-induced (indicating that the promoter is not activated and the protein is not being actively produced); lane 2: induced (indicating that the promoter is activated by IPTG and the protein is actively being produced); lane 3: prewash, lanes 4 and 5 wash 1 and 2; lane 6: elute (purified recombinant chimeric antigen); lane C: control protein (recombinant nucleocapsid (N) protein of SARS-CoV-2).

The chimeric gene was generated and inserted into a pET23a (+) vector. The optimized DNA sequences of SsIR-Ss1a (873 bp) were successfully cloned into competent *E*. *coli* DH5α cells. The protein was purified using a Ni-NTA column at a concentration of 1 mg/ml. SDS-PAGE evaluation verified the purity and molecular weight (MW) of the chimeric antigen (SsIR-Ss1a: 35 kDa) (Fig 4). It is worth mentioning that a 35kDa band in lane 1 can be seen. The reason for having this band is that many promoters show leakiness in their expression i.e. gene products are already expressed at a low level before the addition of the inducer and the presence of this band in the non-inducing line is a result of leakage in the pEt23a vector. pET 23a contains T7 promoter but does not have Lac I gene for synthesizing the lac repressor, LacI. The lacI protein, effectively decreases the level of gene expression prior to inducing IPTG.

**Fig 5:**
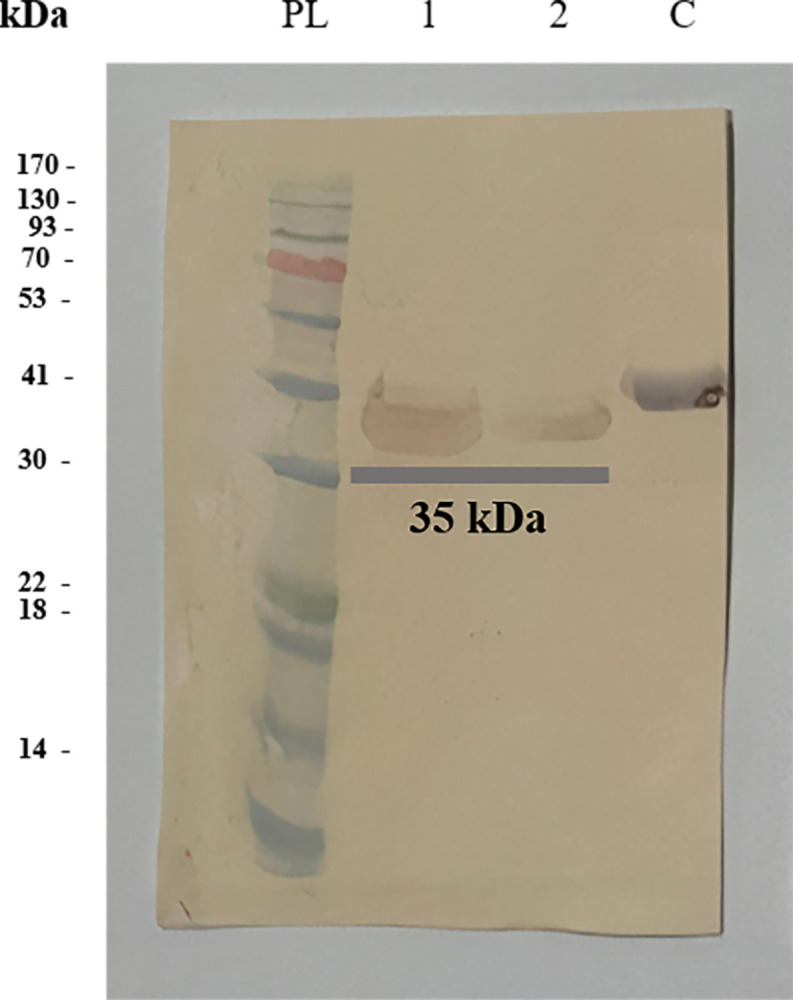
Western blotting analysis of the recombinant chimeric antigen. PL: protein Ladder, lanes 1 and 2: elute (purified recombinant chimeric antigen); lane C: control protein (recombinant nucleocapsid (N) protein of SARS-CoV-2).

Additionally, western blotting analysis confirmed the uniqueness of the protein (Fig 5).

### Evaluation of the diagnostic performance of rSsIR-Ss1a*-*ELISA

**Fig 6:**
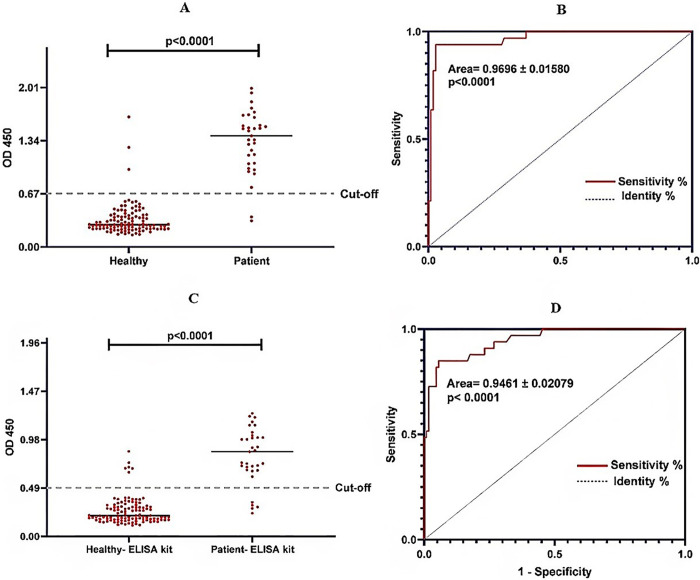
Dot plot result and ROC analysis of rSsIR-Ss1a*-*ELISA. (A) Dot plot result of rSsIR-Ss1a***-***ELISA with chimeric antigen. (B) ROC analysis of rSsIR-Ss1a***-***ELISA with chimeric antigen. (C) Dot plot result of rSsIR-Ss1a***-***ELISA with ELISA kit. (D) ROC analysis of rSsIR-Ss1a***-***ELISA with ELISA kit.

Using the optimum cutoff value (0.6705) calculated by Youden’s J statistic, 31 out of 33 samples from individuals who tested positive for strongyloidiasis through parasitological or PCR methods, were positive by the developed rSsIR-Ss1a***-***ELISA while two of them were false negatives (S3, Optical Density of the samples in the ELISA system). All 56 serum samples of healthy people were negative by the rSsIR-Ss1a***-***ELISA whereas cross-reactivity with the sera of patients with toxocariasis (N = 2) and hydatidosis (N = 1) was observed (Table 3). The sensitivity and specificity of the rSsIR-Ss1a-ELISA, were found to be 93.94% (95% CI, 0.803 to 0.989) and 97.22% (95% CI, 0.921 to 0.992) respectively (Table 4). Due to the non-normal distribution of optical absorption in the serum, we employed the Mann-Whitney U test to evaluate the significance of differences in the mean optical absorption between the patient and control groups. A significant difference was observed, with the patient group showing the mean optical absorption of 1.32±0.39 compared to 0.34±0.19 in the control group (p < 0.05) (S2 Table). The area under curve (AUC) in receiver operating characteristic curve (ROC) analysis was 0.969±0.016 (Fig 6).

**Table 3:** Evaluation of the sera of the patients with various parasitic diseases with rSsIR-Ss1a-ELISA and commercial ELISA Kit.

Type of other infection and autoimmune disease (Cross-reactivity)	N	rSsIR-Ss1a-ELISA		Commercial ELISA Kit	
		Positive	positivity %	Positive	positivity %
**Toxocariasis**	12	2	16.6	3	25
**Fascioliasis**	8	0	0	1	12.5
**Hydatidosis**	8	1	12.5	2	25
**Malaria**	4	0	0	0	0
**Autoimmune disease**	4	0	0	0	0
**Hymenolepiasis**	1	0	0	0	0
**Visceral leishmaniasis**	1	0	0	0	0
**Toxoplasmosis**	3	0	0	0	0
**Cryptosporidiosis**	1	0	0	0	0
**Giardiasis**	2	0	0	0	0
**Trichostrongylosis**	5	0	0	0	0
**FUO (Fever of Unknown Origin)**	3	0	0	0	0

**Table 4:** Sensitivity and specificity for chimeric antigen and ELISA commercial Kit.

	Youden value	Cut-off value	Patient		Healthy		Sensitivity% [95% CI]	Specificity% [95% CI]
			TP	FN	TN	FP		
**Chimeric Antigen**	0.9116	> 0.6705	31	2	105	3	93.94 [80.39 to 98.92]	97.22 [92.15 to 99.24]
**ELISA Kit**	0.7929	> 0.4990	28	5	102	6	84.85 [69.08 to 93.35]	94.44 [88.41 to 97.43]

**Abbreviation:** True Positives (TP); True Negatives (TN); False Positives (FP); False Negatives (FN).

### Performance of commercial ELISA kit

The mean OD of patient sera was 0.81±0.28, while that of healthy sera was 0.25±0.13 (Table 4). A sensitivity of 84.85% (95% CI, 0.690 to 0.933) and a specificity of 94.44% (95% CI, 0.884 to 0.974) were calculated for the commercial kit for the diagnosis of human strongyloidiasis (Table 4). Cross-reactivity was observed with six helminthic infections including toxocariasis (N = 3), hydatidosis (N = 2), and fascioliasis (N = 1) (Table 3).

### Comparison of the results of the commercial kit and experimental ELISA based on rSsIR-Ss1a antigen

Analyzing the diagnostic performance of ELISA tests revealed that the recombinant antigen had higher sensitivity compared to the commercial kit (93.94% vs. 84.85%) (Table 4). Additionally, the specificity of the experimental antigen was higher than that of the commercial kit (97.22 vs. 94.44%) (Table 4). Based on the statistical analysis of the data, there was a good agreement (kappa = 0.83) between the ELISA systems using the produced rSsIR-Ss1a chimeric antigen and the commercial kit (S1 Table).

## Indirect Western Blotting using chimeric antigen (rSsIR-Ss1a)

**Fig 7:**
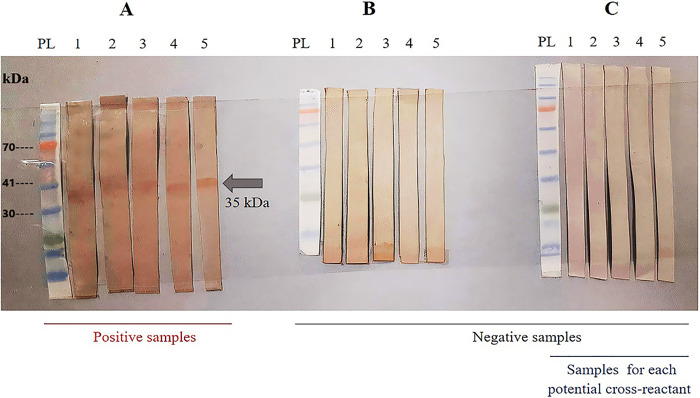
Western blotting of chimeric recombinant antigen (SsIr-Ss1a: 35 KDa), using positive, and negative serums sera. (A), PL: protein size marker, strips 1–5: Strongyloidiasis positive sera; (B), PL: protein size marker, strips 1–5: Negative strongyloidiasis sera; (C), PL: protein size marker, strip 1: pooled toxocariasis sera, strip 2: pooled fascioliasis sera, strip 3: pooled hydatidosis sera, strip 4: pooled trichostrongylosis sera, and strip 5: hymenolepiasis serum.

Western blotting verified the immunoreactivity of the produced chimeric recombinant antigen with the sera of strongyloidiasis patients as shown in Fig 7.

Upon evaluating the cross-reactivity of the produced antigen, using a pool serum in western blotting, no band was seen whereas in ELISA a few positive results were seen. The reason for that in unclear while lower level of antibodies in the pooled serum (as lower amount of each serum is used) or higher sensitivity of the ELISA system can be attributed to these differences.

## Discussion

Strongyloidiasis is a neglected tropical disease that can cause lifelong infection and mortality if not effectively treated. One way to improve the disease control measurements is by developing better diagnostics, particularly through serological approaches [31]. Serological assays are commonly used for the diagnosis of strongyloidiasis, but the available diagnostic tests have limitations in terms of sensitivity and specificity. Therefore, the development of novel diagnostic tools for strongyloidiasis is crucial for the timely and accurate diagnosis of the disease.

In the current study, we designed a chimeric recombinant antigen using immunoinformatics on two reliable parasite antigens, SsIR and Ss1a. To optimize the expression efficiency of our chimeric antigen, we employed codon optimization and in-silico cloning techniques. These strategies ensured efficient expression and enhanced protein production. The produced chimeric antigen (SsIR-Ss1a) was evaluated in an indirect ELISA system to determine its sensitivity and specificity. The results revealed a sensitivity of 93.94% and a specificity of 97.22%. These values indicate that our chimeric antigen has promising performance in detecting strongyloidiasis cases while minimizing false-positive results.

The SsIR-Ss1a chimeric antigen developed in this study is a fusion protein consisting of two *S*. *stercoralis* antigens. The antigens involved in this study, SsIR and Ss1a, have immune-evading properties for parasites [32] and are immunoreactive proteins with an immunomodulatory function [33].

For serological diagnosis, the suitable antigen needs to contain maximum immunogenic regions and minimal overlap with antigens from other infectious organisms [34]. Immunoinformatics approaches help us exclude cross-reactive and focus on highly immunogenic regions. Here, with the help of bioinformatics methods, we designed the intended chimeric antigen, which has good scores in terms of physicochemical properties, antigenicity, cross-reaction, and 3D structure. The antigen was successfully expressed at the applicable concentration.

Until now, several recombinant antigens have been introduced for the serodiagnosis of strongyloidiasis. Among these, the 31-kDa recombinant antigen (NIE) and immunoreactive antigen (SsIR) showed high performance and are available as commercial kits [16].

Recent studies on the development and evaluation of diagnostic tests for strongyloidiasis have shown varying levels of sensitivity and specificity. Arifin et al. developed a novel recombinant Ss-1a protein from the *S*. *stercoralis* genome, showing a sensitivity of 96% and specificity of 93% using ELISA, suggesting it may outperform the NIE antigen in serodiagnosis of strongyloidiasis [32]. Boonroumkaew et al. developed rapid tests based on the SsIR antigen, achieving sensitivities of 91.7% for IgG and 78.3% for IgG4, with specificities of 83.8% and 84.8%, respectively [35]. Tamarozzi et al. evaluated an immunochromatographic test (ICT) and an ELISA using NIE/SsIR recombinant antigens, with the ICT showing lower performance compared to the ELISA, which demonstrated sensitivities up to 92% for IgG and specificities up to 94% for IgG4 [36]. Sears et al. reported high sensitivities and specificities (up to 99% and 100%, respectively) using a prototype recombinant Ss-NIE/Ss-IR-based ELISA [37]. Ahmad et al. introduced a novel recombinant protein, rA133, for an IgE-based ELISA, achieving a sensitivity of 100% and specificity of 99.3% [38]. Additionally, De Souza et al. and Rascoe et al. utilized recombinant antigens in various assay formats, consistently showing high sensitivities and specificities [39,40]. These findings underscore the ongoing efforts to refine serological diagnostics for strongyloidiasis, focusing on improving sensitivity and specificity through various antigen formulations.

Upon comparing the performances of our produced chimeric antigens with those of previously reported antigens, it seems that our developed SsIR-Ss1a ELISA exhibits comparable or even superior sensitivity and specificity values. This suggests that our chimeric recombinant antigen holds promise as an effective diagnostic tool for human strongyloidiasis.

In the current study, despite designing the antigen based on immunoinformatics and identifying common areas between *S*. *stercoralis* and other helminthic parasites, cross-reaction with toxocariasis and hydatidosis sera samples was observed. Previous studies conducted in Iran have indicated a significant seroprevalence of hydatidosis and toxocariasis [41,42]. If we do not consider the possibility of simultaneous infection in these subjects, the cause of cross-reactivity remains unclear. Cross-reactivity in serological assays occurs when antibodies bind to non-target antigens, leading to false-positive or false-negative results. This can be due to structural similarities between antigens from different pathogens or polyclonal antibody responses with low specificity. Understanding these causes is essential for accurate interpretation and improved specificity of serological tests.

The present study has several limitations. Our study utilized a limited panel of sera samples, which restricts the robustness of our findings. To fully assess the performance of the developed SsIR-Ss1a ELISA system, it is crucial to test it using a larger sample size and a diverse control panel from individuals with various parasitic infections, particularly helminthic diseases, as well as samples from patients with conditions that may be misdiagnosed as strongyloidiasis. Additionally, samples were collected from areas where *S*. *stercoralis* is not endemic, reducing the likelihood of co-infection. Due to restricted access to certain helminth infections, we were compelled to limit our sera samples to a few helminthic infections for evaluating cross-reactivity. Possible co-infection was not excluded in all samples which should be considered as another limitation of this study. Also, the study design may have led to an overestimation of the sensitivity and specificity of the assay. This is a common issue in preliminary studies and highlights the need for further validation with a more extensive and varied cohort.

## Conclusion

In the current study, a chimeric recombinant antigen was designed using two parasite antigens, SsIR and Ss1a. The solubility of the antigen was confirmed through physicochemical properties analysis, and the selected regions demonstrated appropriate antigenicity scores. NCBI BLAST analysis of chimeric antigen revealed no commonalities with other parasitic agents. Efficient expression and satisfactory protein purification were achieved through codon optimization and in-silico cloning techniques. The rSsIR-Ss1a-ELISA system exhibited high sensitivity (93.94%) and specificity (97.22%), comparable or superior to what previously published about NIE/SsIR or rSs-NIE-1 antigens in the serodiagnosis of strongyloidiasis. The preliminary findings of our study present a promising evaluation of the novel chimeric recombinant antigen SsIR-Ss1a for the serodiagnosis of human strongyloidiasis. Despite being based on a limited panel of samples, the results indicate potential utility, necessitating further research with larger sample sizes to confirm its diagnostic performance.

## Supporting information

S1 STARD Checklist STARD checklist for reporting of studies of diagnostic accuracy. (DOC)

S1 Data Sequence of the cloned antigen (rSsIR-Ss1a). (DOCX)

S1 Table Optical Density of the samples in the ELISA system. (DOCX)

S2 Table SPSS file of the statistical analysis of data. (DOCX)
