# Correction: Design and expression of a chimeric recombinant antigen (SsIR-Ss1a) for the serodiagnosis of human strongyloidiasis: Evaluation of performance, sensitivity, and specificity

**DOI:** 10.1371/journal.pntd.0012838

**Published:** 2025-01-29

**Authors:** Mostafa Omidian, Zohreh Mostafavi-Pour, Marzieh Asadi, Meysam Sharifdini, Navid Nezafat, Ali Pouryousef, Amir Savardashtaki, Mortaza Taheri-Anganeh, Fattaneh Mikaeili, Bahador Sarkari

In the Introduction of this article [1], a citation [2] (reference 32 of [1]) is omitted from the first sentence of the fourth paragraph.

The correct sentence is: So far, several recombinant antigens have been produced and evaluated for the diagnosis of strongyloidiasis, of which a 31-kDa recombinant antigen (NIE), *S*. *stercoralis* immunoreactive antigen (SsIR), fatty acid and retinol-binding protein (FAR), 14-3-3 protein, and the recently introduced Ss1a-recombinant protein have received more attention [17–20, 32].

The reference is: Arifin N, Yunus MH, Nolan TJ, Lok JB, Noordin R (2018) Identification and preliminary evaluation of a novel recombinant protein for serodiagnosis of strongyloidiasis. The American Journal of Tropical Medicine and Hygiene. 98(4):1165–1170. doi: 10.4269/ajtmh.17-0697
