# Supplementary material for: Design and expression of a chimeric recombinant antigen (SsIR-Ss1a) for the serodiagnosis of human strongyloidiasis: Evaluation of performance, sensitivity, and specificity
Source: PLoS Negl Trop Dis. 2024 Jul 15;18(7):e0012320. doi: 10.1371/journal.pntd.0012320 (PMC11271862; doi:10.1371/journal.pntd.0012320)
Supplement: S1 STARD Checklist — (DOC) [file pntd.0012320.s001.doc]

# STARD checklist for reporting of studies of diagnostic accuracy

*(version 2015)*

***Line numbered against the clean manuscript***

| **Section and Topic** | **No** | **Item** | **On line/paragraph/section** |
| --- | --- | --- | --- |
| **Title or abstract** | | | |
|  | 1 | Identification as a study of diagnostic accuracy using at least one measure of accuracy (such as sensitivity, specificity, predictive values, or AUC) | Title of the study |
| **Abstract** | | |  |
|  | 2 | Structured summary of study design, methods, results, and conclusions (for specific guidance, see STARD for Abstracts) | Abstract of the manuscript |
| **Introduction** | | | |
|  | 3 | Scientific and clinical background, including the intended use and clinical role of the index test | Introduction, the last paragraph |
|  | 4 | Study objectives and hypotheses. | Introduction, the last paragraph |
| **Methods** | | | |
| **Study design** | 5 | Whether data collection was planned before the index test and reference standard were performed (prospective study) or after (retrospective study) | Methods/ sub heading of Serum Sampling |
| **Participants** | 6 | Eligibility criteria | Methods/Serum Sampling sub section |
|  | 7 | On what basis potentially eligible participants were identified (such as symptoms, results from previous tests, inclusion in registry) | Methods/Serum Sampling sub section |
|  | 8 | Where and when potentially eligible participants were identified (setting, location, and dates) | Methods/Serum Sampling sub section |
|  | 9 | Whether participants formed a consecutive, random, or convenience series |  |
| **Test methods** | 10a | Index test, in sufficient detail to allow replication | Methods/ Evaluation of the samples by commercial ELISA Kit |
|  | 10b | Reference standard, in sufficient detail to allow replication |  |
|  | 11 | Rationale for choosing the reference standard (if alternatives exist) | Methods/Serum sampling |
|  | 12a | Definition of and rationale for test positivity cut-offs or result categories of the index test, distinguishing pre-specified from exploratory | Results/Evaluation of the diagnostic performance of rssir-Ss1a-ELISA sub section |
|  | 12b | Definition of and rationale for test positivity cut-offs or result categories of the reference standard, distinguishing pre-specified from exploratory |  |
|  | 13a | Whether clinical information and reference standard results were available to the performers or readers of the index test | Yes. Methods/Serum Sampling subheading |
|  | 13b | Whether clinical information and index test results were available to the assessors of the reference standard | Yes |
| **Analysis** | 14 | Methods for estimating or comparing measures of diagnostic accuracy | Methods/ subheading of Statistical analysis |
|  | 15 | How indeterminate index test or reference standard results were handled | Methods/Serum Sampling sub section |
|  | 16 | How missing data on the index test and reference standard were handled | There is no missing data on the index test |
|  | 17 | Any analyses of variability in diagnostic accuracy, distinguishing pre-specified from exploratory | Results/Evaluation of the diagnostic performance of rssir-Ss1a-ELISA subsection |
|  | 18 | Intended sample size and how it was determined | Methods/Serum Sampling sub section |
| **Results** | | | |
| **Participants** | 19 | Flow of participants, using a diagram |  |
|  | 20 | Baseline demographic and clinical characteristics of participants | Methods/Serum Sampling sub- section |
|  | 21a | Distribution of severity of disease in those with the target condition |  |
|  | 21b | Distribution of alternative diagnoses in those without the target condition |  |
|  | 22 | Time interval and any clinical interventions between index test and reference standard | Methods/Serum Sampling sub section/ |
| **Test results** | 23 | Cross tabulation of the index test results (or their distribution) by the results of the reference standard | Result section, Table 4, |
|  | 24 | Estimates of diagnostic accuracy and their precision (such as 95% confidence intervals) | Result section, Table 4 |
|  | 25 | Any adverse events from performing the index test or the reference standard |  |
| **Discussion** | | | |
|  | 26 | Study limitations, including sources of potential bias, statistical uncertainty, and generalisability | Discussion section |
|  | 27 | Implications for practice, including the intended use and clinical role of the index test | Discussion /Conclusion section |
| **Other information** | | | |
|  | 28 | Registration number and name of registry |  |
|  | 29 | Where the full study protocol can be accessed | Within the manuscript |
|  | 30 | Sources of funding and other support; role of funders | Source of funding was provided in the related section. |
