# Supplementary material for: Design and expression of a chimeric recombinant antigen (SsIR-Ss1a) for the serodiagnosis of human strongyloidiasis: Evaluation of performance, sensitivity, and specificity
Source: PLoS Negl Trop Dis. 2024 Jul 15;18(7):e0012320. doi: 10.1371/journal.pntd.0012320 (PMC11271862; doi:10.1371/journal.pntd.0012320)
Supplement: S1 Data — (DOCX) [file pntd.0012320.s002.docx]

10 20 30 40 50 60 70 80 90 100 110


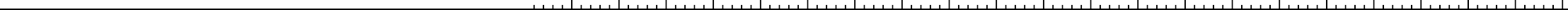


D04-W383056-C9228IA300-2-pET-seqR.ab1(1>834) C9228IA300-2.seq(1>885)

D04-W383056-C9228IA300-2-pET-seqR.ab1(1>834) C9228IA300-2.seq(1>885)

D04-W383056-C9228IA300-2-pET-seqR.ab1(1>834) C9228IA300-2.seq(1>885)

D02-W383056-C9228IA300-2-2-SEQ1F.ab1(1>758)

D04-W383056-C9228IA300-2-pET-seqR.ab1(1>834) C9228IA300-2.seq(1>885)

D02-W383056-C9228IA300-2-2-SEQ1F.ab1(1>758)

D04-W383056-C9228IA300-2-pET-seqR.ab1(1>834) C9228IA300-2.seq(1>885)

D02-W383056-C9228IA300-2-2-SEQ1F.ab1(1>758)

D04-W383056-C9228IA300-2-pET-seqR.ab1(1>834) C9228IA300-2.seq(1>885)

D02-W383056-C9228IA300-2-2-SEQ1F.ab1(1>758)

D04-W383056-C9228IA300-2-pET-seqR.ab1(1>834) C9228IA300-2.seq(1>885)

D02-W383056-C9228IA300-2-2-SEQ1F.ab1(1>758)

D04-W383056-C9228IA300-2-pET-seqR.ab1(1>834) C9228IA300-2.seq(1>885)

D02-W383056-C9228IA300-2-2-SEQ1F.ab1(1>758)

TAATTTTGTTTAACTTTAAGAAGGAGATATACATATGAACAGCGCGCGTGTGGAAAATCAGGATCAGAAGGATCAGCTGGAGAACCAGGACCAAAAAGATCAACTGGAAA


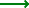

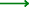
TAATTTTGTTTAACTTTAAGAAGGAGATATACATATGAACAGCgCgCGTGtGGAAAATCAGGATCAGAAGGATCAGCTGGAGAACCaGGACCAAAAAGATCAACTGGAAA CATATGAACAGCGCGCGTGTGGAAAATCAGGATCAGAAGGATCAGCTGGAGAACCAGGACCAAAAAGATCAACTGGAAA


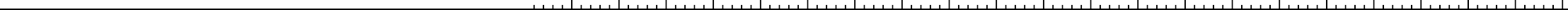
120 130 140 150 160 170 180 190 200 210 220

ACCAGGACCAGAAGAACCAGCTGAAGAACCAAAGCGAGAACCAGGATCAGAAGAACCAACTGAAAAACCAAAGCGAAAATCAAGACCAAAAGAAACCGATCAAGAAACCG


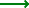

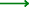
ACCAGGACCAGAAGAACCAGCTGAAGAACCAAAGCGAGAACCAGGATCAGAAGAACCAACTGAAAAACCAAAGCGAAAATCAAGACCAAAAGAAACCGATCAAGAAACCG ACCAGGACCAGAAGAACCAGCTGAAGAACCAAAGCGAGAACCAGGATCAGAAGAACCAACTGAAAAACCAAAGCGAAAATCAAGACCAAAAGAAACCGATCAAGAAACCG


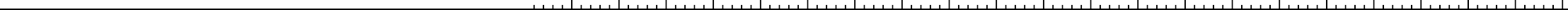
230 240 250 260 270 280 290 300 310 320 330

ATTAAGAAACCGGGTCCGAAGCCGATCCGTCCGATTGTGAAGCCGAAACCGAAGACCACCACCCAAGCGCCGGAGGAACCGGAGGGTCCGGAGGAACCGGAAGGTCCGGA


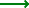

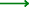
ATTAAGAAACCGGGTCCGAAGCCGATCCGTCCGATTGTGAAGCCGAAACCGAAGACCACCACCCAAGCGCCGGAGGAACCGGAGGGTCCGGAGGAACCGGAAGGTCCGGA ATTAAGAAACCGGGTCCGAAGCCGATCCGTCCGATTGTGAAGCCGAAACCGAAGACCACCACCCAAGCGCCGGAGGAACCGGAGGGTCCGGAGGAACCGGAAGGTCCGGA


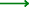
GTGAAGCCGAAACCGAAGACCACCACCCAAGCGCCGGAGGAACCGGAGGGTCCGGAGGAACCGGAAGGTCCGGA


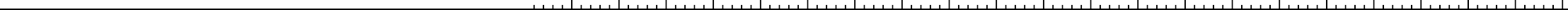
340 350 360 370 380 390 400 410 420 430 440

GGCGGCGGCGAAAAACCTGCACCTGTACTATATCATTGACTTCGTTCTGCCGTGGCTGAAAGATAAAGAGGAGAGCAGCAGCGGCCCGAGCATCAGCAAAGACGATAAGC


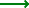

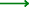

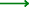
GGCGGCGGCGAAAAACCTGCACCTGTACTATATCATTGACTTCGTTCTGCCGTGGCTGAAAGATAAAGAGGAGAGCAGCAGCGGCCCGAGCATCAGCAAAGACGATAAGC GGCGGCGGCGAAAAACCTGCACCTGTACTATATCATTGACTTCGTTCTGCCGTGGCTGAAAGATAAAGAGGAGAGCAGCAGCGGCCCGAGCATCAGCAAAGACGATAAGC GGCGGCGGCGAAAAACCTGCACCTGTACTATATCATTGACTTCGTTCTGCCGTGGCTGAAAGATAAAGAGGAGAGCAGCAGCGGCCCGAGCATCAGCAAAGACGATAAGC


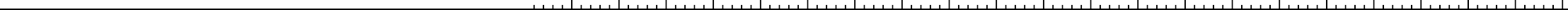
450 460 470 480 490 500 510 520 530 540 550

TGACCCCGAGCGAACGTCGTGAGCGTATTCTGAAGCGTCACCAGATGTACAAAAACTTTGAGGAAAAGCTGCTGGAGTATGAAAACGAGGCGAGCACCGCGGGTGGCCTG


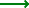

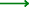

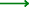
TGACCCCGAGCGAACGTCGTGAGCGTATTCTGAAGCGTCACCAGATGTACAAAAACTTTGAGGAAAAGCTGCTGGAGTATGAAAACGAGGCGAGCACCGCGGGTGGCCTG TGACCCCGAGCGAACGTCGTGAGCGTATTCTGAAGCGTCACCAGATGTACAAAAACTTTGAGGAAAAGCTGCTGGAGTATGAAAACGAGGCGAGCACCGCGGGTGGCCTG TGACCCCGAGCGAACGTCGTGAGCGTATTCTGAAGCGTCACCAGATGTACAAAAACTTTGAGGAAAAGCTGCTGGAGTATGAAAACGAGGCGAGCACCGCGGGTGGCCTG


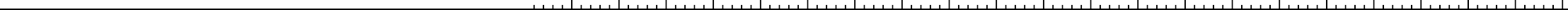
560 570 580 590 600 610 620 630 640 650 660

GACGATATCACCCAACGTAACTACGTGCTGGCGAAACTGCGTACCTATGCGCTGAAGGCGATGATGGATCTGGAAAAAATCGGCGAGGAACTGGGCATTCTGGAGTACAT


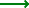

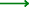

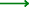
GACGATATCACCCAACGTAACTACGTGCTGGCGAAACTGCGTACCTATGCGCTGAAGGCGATGATGGATCTGGAAAAAATCGGCGAGGAACTGGGCATTCTGGAGTACAT GACGATATCACCCAACGTAACTACGTGCTGGCGAAACTGCGTACCTATGCGCTGAAGGCGATGATGGATCTGGAAAAAATCGGCGAGGAACTGGGCATTCTGGAGTACAT GACGATATCACCCAACGTAACTACGTGCTGGCGAAACTGCGTACCTATGCGCTGAAGGCGATGATGGATCTGGAAAAAATCGGCGAGGAACTGGGCATTCTGGAGTACAT


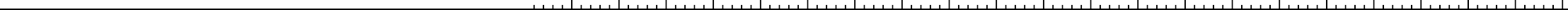
670 680 690 700 710 720 730 740 750 760 770

GCTGAAAATCAAGCAGGGTGAAGTGGTTGAGGAAAAACACAAGCCGCCGCCGAAGATGACCACCTATCGTATTGTGCGTAACGAGGAACAGAAGAAAAGCCTGGAGTGGG


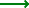

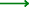

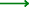
GCTGAAAATCAAGCAGGGTGAAGTGGTTGAGGAAAAACACAAGCCGCCGCCGAAGATGACCACCTATCGTATTGTGCGTAACGAGGAACAGAAGAAAAGCCTGGAGTGGG GCTGAAAATCAAGCAGGGTGAAGTGGTTGAGGAAAAACACAAGCCGCCGCCGAAGATGACCACCTATCGTATTGTGCGTAACGAGGAACAGAAGAAAAGCCTGGAGTGGG GCTGAAAATCAAGCAGGGTGAAGTGGTTGAGGAAAAACACAAGCCGCCGCCGAAGATGACCACCTATCGTATTGTGCGTAACGAGGAACAGAAGAAAAGCCTGGAGTGGG


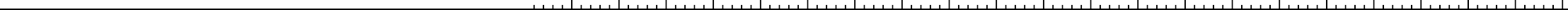
780 790 800 810 820 830 840 850 860 870 880

TTATCAAAATTTTCCAACACCTGCTGTGGATGAGCGGCATCGTTAAATGGATTCAGAAGGACATCCTGATTCTGAACCGTACCCCGGAACACAGCCCGATCCCGCAAATT


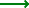

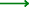

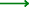
TTATCAAAATTTTCCAACACCTGCTGTGGATGAGCGGCATCGTTAAATGGATTCAGAAGGACAT TTATCAAAATTTTCCAACACCTGCTGTGGATGAGCGGCATCGTTAAATGGATTCAGAAGGACATCCTGATTCTGAACCGTACCCCGGAACACAGCCCGATCCCGCAAATT TTATCAAAATTTTCCAACACCTGCTGTGGATGAGCGGCATCGTTAAATGGATTCAGAAGGACATCCTGATTCTGAACCGTACCCCGGAACACAGCCCGATCCCGCAAATT

890 900 910 920 930 940 950 960 970 980 990

C9228IA300-2.seq(1>885)

D02-W383056-C9228IA300-2-2-SEQ1F.ab1(1>758)

D02-W383056-C9228IA300-2-2-SEQ1F.ab1(1>758)

ATGGAGGAAACCATGATGATGATGATCATTCTCGAGCACCACCACCACCACCACTGAGATCCGGCTGCTAACAAAGCCCGAAAGGAAGCTGAGTTGGCTGCTGCCACCGC

ATGGAGGAAACCATGATGATGATGATCATTCTCGAG ATGGAGGAAACCATGATGATGATGATCATTCTCGAGCACCACCACCACCACCACTGAGATCCGGCTGCTAACAAAGCCCGAAAGGAAGCTGAGTTGGCTGCTGCCACCGC

1000 1010

TGAGCAATAACTAGCATAACCCCT TGAGCAATAACTAGCATAACCCCT
