# Supplementary material for: Design and expression of a chimeric recombinant antigen (SsIR-Ss1a) for the serodiagnosis of human strongyloidiasis: Evaluation of performance, sensitivity, and specificity
Source: PLoS Negl Trop Dis. 2024 Jul 15;18(7):e0012320. doi: 10.1371/journal.pntd.0012320 (PMC11271862; doi:10.1371/journal.pntd.0012320)
Supplement: S1 Table — (DOCX) [file pntd.0012320.s003.docx]

**Contents:**

**Table 1: Optical density of Positive samples …………………….………………………………..…**i

**Table 2: Optical density of Negative samples………..………………………...……………………**ii

**Table 3: Optical density of other parasitic disease samples……………………………...………** iv

**Abbreviations: True positive (TP); False negative (FN)**

**Table 1: OD of positive sera for strongyloidiasis in Ss/Ir-Ss1a ELISA and commercial ELISA Kit**

| **Ss/Ir-Ss1a ELISA [TP: 31 / FN: 2]** | | | | **ELISA Kit [TP: 28 / FN: 5]** | | |
| --- | --- | --- | --- | --- | --- | --- |
| **Number** | **OD** | **Result** | | **OD** | **Results** | |
| **1** | 0.922 | + | TP | 0.718 | + | TP |
| **2** | 1.492 | + | TP | 0.674 | + | TP |
| **3** | 1.83 | + | TP | 0.739 | + | TP |
| **4** | 0.751 | + | TP | 0.346 | - | FN |
| **5** | 1.055 | + | TP | 0.662 | + | TP |
| **6** | 1.218 | + | TP | 0.746 | + | TP |
| **7** | 2 | + | TP | 1.000 | + | TP |
| **8** | 1.464 | + | TP | 0.316 | - | FN |
| **9** | 1.942 | + | TP | 1.207 | + | TP |
| **10** | 1.517 | + | TP | 0.737 | + | TP |
| **11** | 1.7 | + | TP | 1.246 | + | TP |
| **12** | 1.349 | + | TP | 0.703 | + | TP |
| **13** | 1.165 | + | TP | 0.298 | - | FN |
| **14** | 1.309 | + | TP | 0.858 | + | TP |
| **15** | 1.293 | + | TP | 0.711 | + | TP |
| **16** | 1.5 | + | TP | 0.906 | + | TP |
| **17** | 1.75 | + | TP | 1.054 | + | TP |
| **18** | 1.4 | + | TP | 0.982 | + | TP |
| **19** | 1.35 | + | TP | 1.024 | + | TP |
| **20** | 0.973 | + | TP | 0.899 | + | TP |
| **21** | 1.496 | + | TP | 0.983 | + | TP |
| **22** | 0.989 | + | TP | 0.606 | + | TP |
| **23** | 1.156 | + | TP | 1.160 | + | TP |
| **24** | 1.533 | + | TP | 0.668 | + | TP |
| **25** | 0.951 | + | TP | 0.716 | + | TP |
| **26** | 1.667 | + | TP | 1.127 | + | TP |
| **27** | 0.330 | - | FN | 0.284 | - | FN |
| **28** | 1.634 | + | TP | 1.127 | + | TP |
| **29** | 0.379 | - | FN | 0.235 | - | FN |
| **30** | 1.05 | + | TP | 1.216 | + | TP |
| **31** | 1.66 | + | TP | 1.000 | + | TP |
| **32** | 1.525 | + | TP | 0.997 | + | TP |
| **33** | 1.49 | + | TP | 0.87 | + | TP |

**Table 2: OD of negative sera for strongyloidiasis in Ss/Ir-Ss1a ELISA and commercial ELISA Kit**

| **Ss/Ir-Ss1a ELISA [TN: 56 / FP: 0]** | | | | **ELISA Kit [TN: 56 / FP: 0]** | | |
| --- | --- | --- | --- | --- | --- | --- |
| **Number** | **OD** | **Results** | | **OD** | **Results** | |
| **1** | 0.310 | - | TN | 0.198 | - | TN |
| **2** | 0.188 | - | TN | 0.351 | - | TN |
| **3** | 0.286 | - | TN | 0.264 | - | TN |
| **4** | 0.265 | - | TN | 0.211 | - | TN |
| **5** | 0.367 | - | TN | 0.303 | - | TN |
| **6** | 0.235 | - | TN | 0.388 | - | TN |
| **7** | 0.297 | - | TN | 0.195 | - | TN |
| **8** | 0.247 | - | TN | 0.296 | - | TN |
| **9** | 0.259 | - | TN | 0.265 | - | TN |
| **10** | 0.265 | - | TN | 0.347 | - | TN |
| **11** | 0.202 | - | TN | 0.154 | - | TN |
| **12** | 0.224 | - | TN | 0.193 | - | TN |
| **13** | 0.271 | - | TN | 0.331 | - | TN |
| **14** | 0.343 | - | TN | 0.280 | - | TN |
| **15** | 0.385 | - | TN | 0.299 | - | TN |
| **16** | 0.360 | - | TN | 0.377 | - | TN |
| **17** | 0.232 | - | TN | 0.320 | - | TN |
| **18** | 0.214 | - | TN | 0.149 | - | TN |
| **19** | 0.193 | - | TN | 0.272 | - | TN |
| **20** | 0.274 | - | TN | 0.356 | - | TN |
| **21** | 0.248 | - | TN | 0.187 | - | TN |
| **22** | 0.224 | - | TN | 0.163 | - | TN |
| **23** | 0.321 | - | TN | 0.116 | - | TN |
| **24** | 0.219 | - | TN | 0.214 | - | TN |
| **25** | 0.338 | - | TN | 0.252 | - | TN |
| **26** | 0.199 | - | TN | 0.294 | - | TN |
| **27** | 0.258 | - | TN | 0.338 | - | TN |
| **28** | 0.262 | - | TN | 0.387 | - | TN |
| **29** | 0.267 | - | TN | 0.392 | - | TN |
| **30** | 0.226 | - | TN | 0.293 | - | TN |
| **31** | 0.190 | - | TN | 0.375 | - | TN |
| **32** | 0.287 | - | TN | 0.192 | - | TN |
| **33** | 0.231 | - | TN | 0.225 | - | TN |
| **34** | 0.261 | - | TN | 0.250 | - | TN |
| **35** | 0.170 | - | TN | 0.148 | - | TN |
| **36** | 0.271 | - | TN | 0.378 | - | TN |
| **37** | 0.313 | - | TN | 0.211 | - | TN |
| **38** | 0.235 | - | TN | 0.278 | - | TN |
| **39** | 0.242 | - | TN | 0.342 | - | TN |
| **40** | 0.287 | - | TN | 0.299 | - | TN |
| **41** | 0.204 | - | TN | 0.208 | - | TN |
| **42** | 0.223 | - | TN | 0.184 | - | TN |
| **43** | 0.160 | - | TN | 0.391 | - | TN |
| **44** | 0.237 | - | TN | 0.169 | - | TN |
| **45** | 0.252 | - | TN | 0.148 | - | TN |
| **46** | 0.159 | - | TN | 0.112 | - | TN |
| **47** | 0.234 | - | TN | 0.127 | - | TN |
| **48** | 0.291 | - | TN | 0.266 | - | TN |
| **49** | 0.231 | - | TN | 0.133 | - | TN |
| **50** | 0.276 | - | TN | 0.144 | - | TN |
| **51** | 0.264 | - | TN | 0.284 | - | TN |
| **52** | 0.228 | - | TN | 0.294 | - | TN |
| **53** | 0.166 | - | TN | 0.317 | - | TN |
| **54** | 0.229 | - | TN | 0.154 | - | TN |
| **55** | 0.212 | - | TN | 0.181 | - | TN |
| **56** | 0.153 | - | TN | 0.235 | - | TN |

**Table 3: OD of sera of patients with other parasitic diseases (except strongyloidiasis) in Ss/Ir-Ss1a ELISA and commercial ELISA Kit**

| **Number** | **Ss/Ir-Ss1a ELISA [TN: 49 / FP: 3]** | | | | **ELISA Kit [TN: 46 / FP: 6]** | | |
| --- | --- | --- | --- | --- | --- | --- | --- |
|  | **Sample** | **OD** | **Results** | | **OD** | **Results** | |
| **1** | Toxocariasis | 0.590 | - | TN | 0.174 | - | TN |
| **2** | Toxocariasis | 0.978 | + | FP | 0.690 | + | FP |
| **3** | Toxocariasis | 0.221 | - | TN | 0.270 | - | TN |
| **4** | Toxocariasis | 0.522 | - | TN | 0.279 | - | TN |
| **5** | Toxocariasis | 0.403 | - | TN | 0.140 | - | TN |
| **6** | Toxocariasis | 0.407 | - | TN | 0.861 | + | FP |
| **7** | Toxocariasis | 0.460 | - | TN | 0.191 | - | TN |
| **8** | Toxocariasis | 0.275 | - | TN | 0.376 | - | TN |
| **9** | Toxocariasis | 0.574 | - | TN | 0.149 | - | TN |
| **10** | Toxocariasis | 0.423 | - | TN | 0.284 | - | TN |
| **11** | Toxocariasis | 0.358 | - | TN | 0.363 | - | TN |
| **12** | Toxocariasis | 1.64 | + | FP | 0.747 | + | FP |
| **13** | Fascioliasis | 0.542 | - | TN | 0.205 | - | TN |
| **14** | Fascioliasis | 0.254 | - | TN | 0.169 | - | TN |
| **15** | Fascioliasis | 0.492 | - | TN | 0.221 | - | TN |
| **16** | Fascioliasis | 0.490 | - | TN | 0.688 | + | FP |
| **17** | Fascioliasis | 0.390 | - | TN | 0.145 | - | TN |
| **18** | Fascioliasis | 0.521 | - | TN | 0.157 | - | TN |
| **19** | Fascioliasis | 0.375 | - | TN | 0.182 | - | TN |
| **20** | Fascioliasis | 0.468 | - | TN | 0.179 | - | TN |
| **21** | Hydatidosis | 0.576 | - | TN | 0.309 | - | TN |
| **22** | Hydatidosis | 0.283 | - | TN | 0.261 | - | TN |
| **23** | Hydatidosis | 1.255 | + | FP | 0.702 | + | FP |
| **24** | Hydatidosis | 0.185 | - | TN | 0.649 | + | FP |
| **25** | Hydatidosis | 0.458 | - | TN | 0.187 | - | TN |
| **26** | Hydatidosis | 0.463 | - | TN | 0.346 | - | TN |
| **27** | Hydatidosis | 0.478 | - | TN | 0.146 | - | TN |
| **28** | Hydatidosis | 0.505 | - | TN | 0.198 | - | TN |
| **29** | Malaria | 0.379 | - | TN | 0.262 | - | TN |
| **30** | Malaria | 0.276 | - | TN | 0.171 | - | TN |
| **31** | Malaria | 0.183 | - | TN | 0.291 | - | TN |
| **32** | malaria | 0.426 | - | TN | 0.175 | - | TN |
| **33** | Autoimmune disease | 0.263 | - | TN | 0.321 | - | TN |
| **34** | Autoimmune disease | 0.232 | - | TN | 0.165 | - | TN |
| **35** | Autoimmune disease | 0.230 | - | TN | 0.200 | - | TN |
| **36** | Autoimmune disease | 0.368 | - | TN | 0.118 | - | TN |
| **37** | Hymenolepiasis | 0.327 | - | TN | 0.161 | - | TN |
| **38** | Visceral leishmaniasis | 0.464 | - | TN | 0.152 | - | TN |
| **39** | Toxoplasmosis | 0.362 | - | TN | 0.123 | - | TN |
| **40** | Toxoplasmosis | 0.397 | - | TN | 0.167 | - | TN |
| **41** | Toxoplasmosis | 0.322 | - | TN | 0.182 | - | TN |
| **42** | Cryptosporidiosis | 0.338 | - | TN | 0.192 | - | TN |
| **43** | Giardiasis | 0.277 | - | TN | 0.166 | - | TN |
| **44** | Giardiasis | 0.513 | - | TN | 0.175 | - | TN |
| **45** | Trichostrongylosis | 0.401 | - | TN | 0.201 | - | TN |
| **46** | Trichostrongylosis | 0.383 | - | TN | 0.196 | - | TN |
| **47** | Trichostrongylosis | 0.326 | - | TN | 0.189 | - | TN |
| **48** | Trichostrongylosis | 0.564 | - | TN | 0.133 | - | TN |
| **49** | Trichostrongylosis | 0.416 | - | TN | 0.172 | - | TN |
| **50** | Fever of Unknown Origin | 0.299 | - | TN | 0.165 | - | TN |
| **51** | Fever of Unknown Origin | 0.214 | - | TN | 0.191 | - | TN |
| **52** | Fever of Unknown Origin | 0.311 | - | TN | 0.178 | - | TN |
