# Supplementary material for: Design and expression of a chimeric recombinant antigen (SsIR-Ss1a) for the serodiagnosis of human strongyloidiasis: Evaluation of performance, sensitivity, and specificity
Source: PLoS Negl Trop Dis. 2024 Jul 15;18(7):e0012320. doi: 10.1371/journal.pntd.0012320 (PMC11271862; doi:10.1371/journal.pntd.0012320)

[**Statistical analysis for recombinanta ntigen……………………………………………………………………………**](#_Toc167872940)

**Mann–Whitney U test………………………………………………………………………………………………………………**

The significance of the difference between the healthy and patient groups.


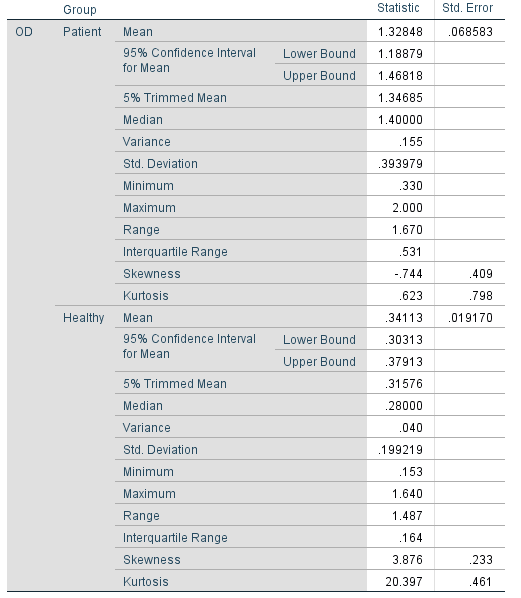


## Statistical analysis for recombinant antigen


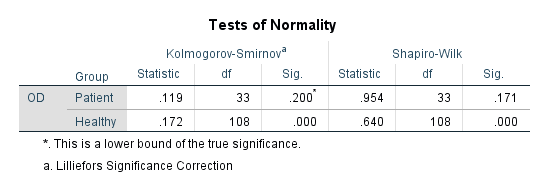
Normality test


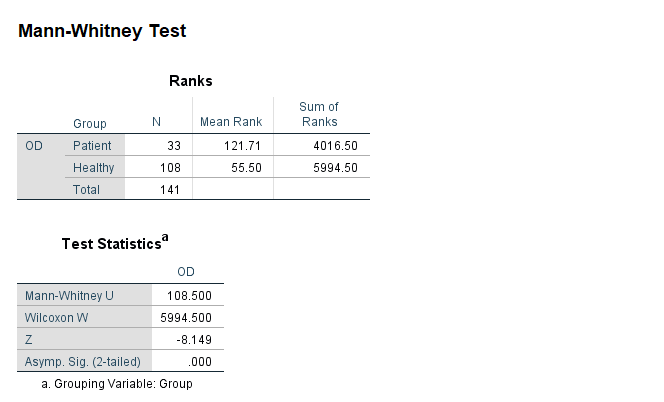


Statistical analysis (SPSS) for ELISA Kit.………………

[Mann–Whitney U test3](#_Toc166406915)

**Statistical analysis (SPSS) for ELISA Kit**


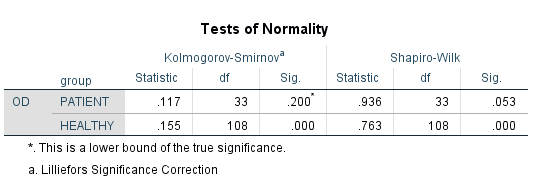

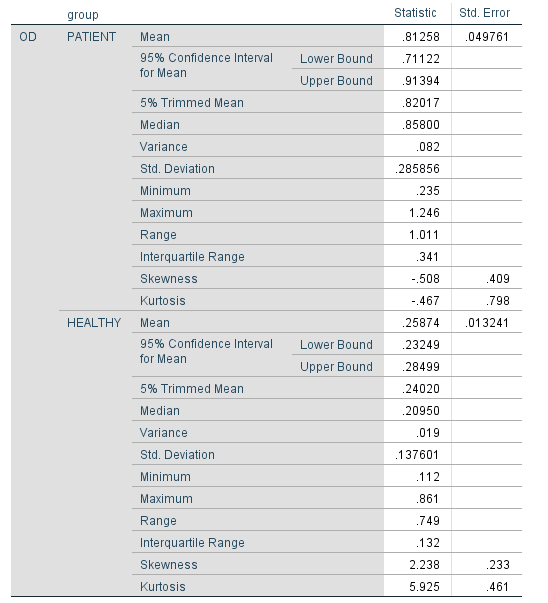


The significance of the difference between the healthy and patient groups.

**Non parametric:** Mann–Whitney U test


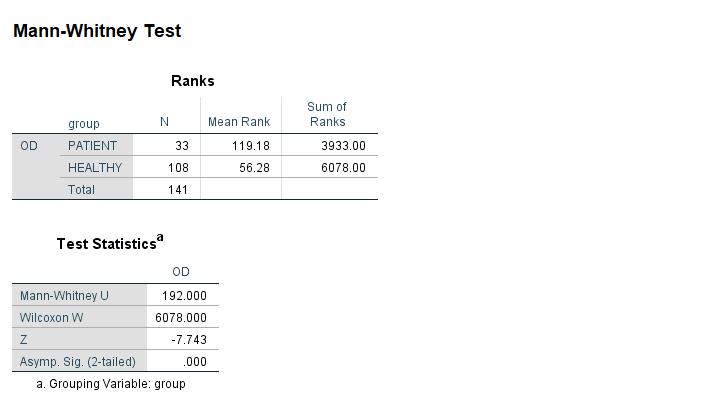

Supplement: S2 Table — (DOCX) [file pntd.0012320.s004.docx]
